# The Neural Mechanisms of Re-Experiencing Mental Fatigue Sensation: A Magnetoencephalography Study

**DOI:** 10.1371/journal.pone.0122455

**Published:** 2015-03-31

**Authors:** Akira Ishii, Takuma Karasuyama, Taiki Kikuchi, Masaaki Tanaka, Emi Yamano, Yasuyoshi Watanabe

**Affiliations:** 1 Department of Physiology, Osaka City University Graduate School of Medicine, 1-4-3 Asahimachi, Abeno-ku, Osaka, 545–8585, Japan; 2 RIKEN, Center for Life Science Technologies, 6-7-3 Minatojima-minamimachi, Chuo-ku, Hyogo, 650–0047, Japan; University of Pennsylvania, UNITED STATES

## Abstract

There have been several studies which have tried to clarify the neural mechanisms of fatigue sensation; however fatigue sensation has multiple aspects. We hypothesized that past experience related to fatigue sensation is an important factor which contributes to future formation of fatigue sensation through the transfer to memories that are located within specific brain structures. Therefore, we aimed to investigate the neural mechanisms of fatigue sensation related to memory. In the present study, we investigated the neural activity caused by re-experiencing the fatigue sensation that had been experienced during a fatigue-inducing session. Thirteen healthy volunteers participated in fatigue and non-fatigue experiments in a crossover fashion. In the fatigue experiment, they performed a 2-back test session for 40 min to induce fatigue sensation, a rest session for 15 min to recover from fatigue, and a magnetoencephalography (MEG) session in which they were asked to re-experience the state of their body with fatigue that they had experienced in the 2-back test session. In the non-fatigue experiment, the participants performed a free session for 15 min, a rest session for 15 min, and an MEG session in which they were asked to re-experience the state of their body without fatigue that they had experienced in the free session. Spatial filtering analyses of oscillatory brain activity showed that the delta band power in the left Brodmann’s area (BA) 39, alpha band power in the right pulvinar nucleus and the left BA 40, and beta band power in the left BA 40 were lower when they re-experienced the fatigue sensation than when they re-experienced the fatigue-free sensation, indicating that these brain regions are related to re-experiencing the fatigue sensation. Our findings may help clarify the neural mechanisms underlying fatigue sensation.

## Introduction

Fatigue is a common problem in modern societies. The Japanese Society of Fatigue Science defined fatigue as a decline in the ability and efficiency of mental and/or physical activities that is caused by excessive mental or physical activities or disease [1]. More than 20–30% of the general population in European countries and the United States experience substantial fatigue [2–6], and in Japan, more than half of the adult population reports experiencing fatigue [7]. Fatigue is often accompanied by a peculiar sense of discomfort, a desire to rest, and a decline in motivation, referred to as fatigue sensation [1]. Fatigue sensation may impair our functioning. Therefore, it is important to clarify the neural mechanisms underlying fatigue sensation to cope with fatigue related problems prevailing in our society. Fatigue can be classified as acute or chronic, and physical or mental. In this study, we focused on the neural mechanisms of fatigue sensation related to acute mental fatigue.

Several studies have tried to clarify the neural mechanisms of fatigue sensation, which has multiple aspects. The level of fatigue sensation induced by performing a prolonged cognitive test was positively correlated with the increase in regional blood flow in the medial orbitofrontal cortex measured using H_2_^15^O positron emission tomography [2]. The self-evaluated level of physical and mental fatigue sensation was related to the change in oscillatory power in the posterior cingulate cortex in magnetoencephalography (MEG) studies [3, 4] and the self-evaluated level of effort required to perform a mental task was related to the neural activation of the insular cortex in a functional magnetic resonance imaging (fMRI) study [5].

One fMRI study asked healthy participants and chronic fatigue syndrome (CFS) patients to imagine themselves in fatiguing situations. The neural activity in the posterior cingulate cortex was higher in CFS patients than in healthy participants, and the neural activity in the dorsolateral prefrontal cortex was lower in CFS patients than in healthy participants [6]. However, in this study, the way in which the participants imagined that they were in a fatiguing situation may have been variable, i.e., they might have recalled autobiographical episodes similar to the situation presented in the experiment or they might have mentally simulated the situation without a specific episode in mind. Although this study showed the effectiveness of a fatigue provocation procedure for investigating the neural mechanisms of fatigue sensation, restricting the way the participants self-induce the fatigue sensation would enable specific aspects of the neural mechanisms of fatigue sensation to be examined and would further increase our understanding of the neural mechanisms related to fatigue sensation.

Emotion-induction paradigms have been successfully utilized to explore the neural substrates of emotions such as anxiety, anger, sadness, disgust, and happiness [7–9]. In an H_2_^15^O positron emission tomography study, the pattern of neural activity caused by self-induced transient sadness during recalling affect-appropriate life events and looking at sad human faces was different from that caused by self-induced transient happiness, indicating that transient sadness and happiness affect different brain regions in divergent directions [7]. An fMRI study of the neural activity caused by symptom provocation in obsessive-compulsive disorder found that a distinct pattern of activation was associated with each symptom of the disorder [10]. Taking these findings into consideration, investigating the neural substrates related to self-induced fatigue sensation could be an effective approach to further the understanding of the neural mechanisms of fatigue sensation in healthy people and patients with fatigue-related diseases such as CFS.

In the present study, we investigated the neural activity induced by re-experiencing a mental fatigue sensation that had actually been experienced by participants during a mental fatigue-inducing session. We hypothesized that past experience related to fatigue sensation is an important factor which contributes to future formation of fatigue sensation through the transfer to memories that are located within specific brain structures and that investigating the neural mechanisms of fatigue sensation related to memory would provide valuable clue to understand the neural mechanisms of fatigue sensation. Therefore, we aimed to investigate the neural mechanisms of fatigue sensation related to memory in healthy participants. The neural activity during re-experiencing the mental fatigue sensation and the sensation without mental fatigue was recorded with high temporal and spatial resolution using MEG to identify the neural activity related to re-experiencing the mental fatigue sensation.

## Materials and Methods

### Participants

Thirteen healthy male volunteers (21.7 ± 3.3 years of age [mean ± SD]) participated in this study. All participants were right-handed according to the Edinburgh Handedness Inventory [11]. Current smokers, individuals with a history of mental or brain disorder, and individuals taking chronic medications that affect the central nervous system were excluded. The Ethics Committee of Osaka City University approved the study protocol (approval number, 2697). All participants provided written informed consent for participation in this study in accordance with the principles of the Declaration of Helsinki.

### Experimental design

**Fig 1:**
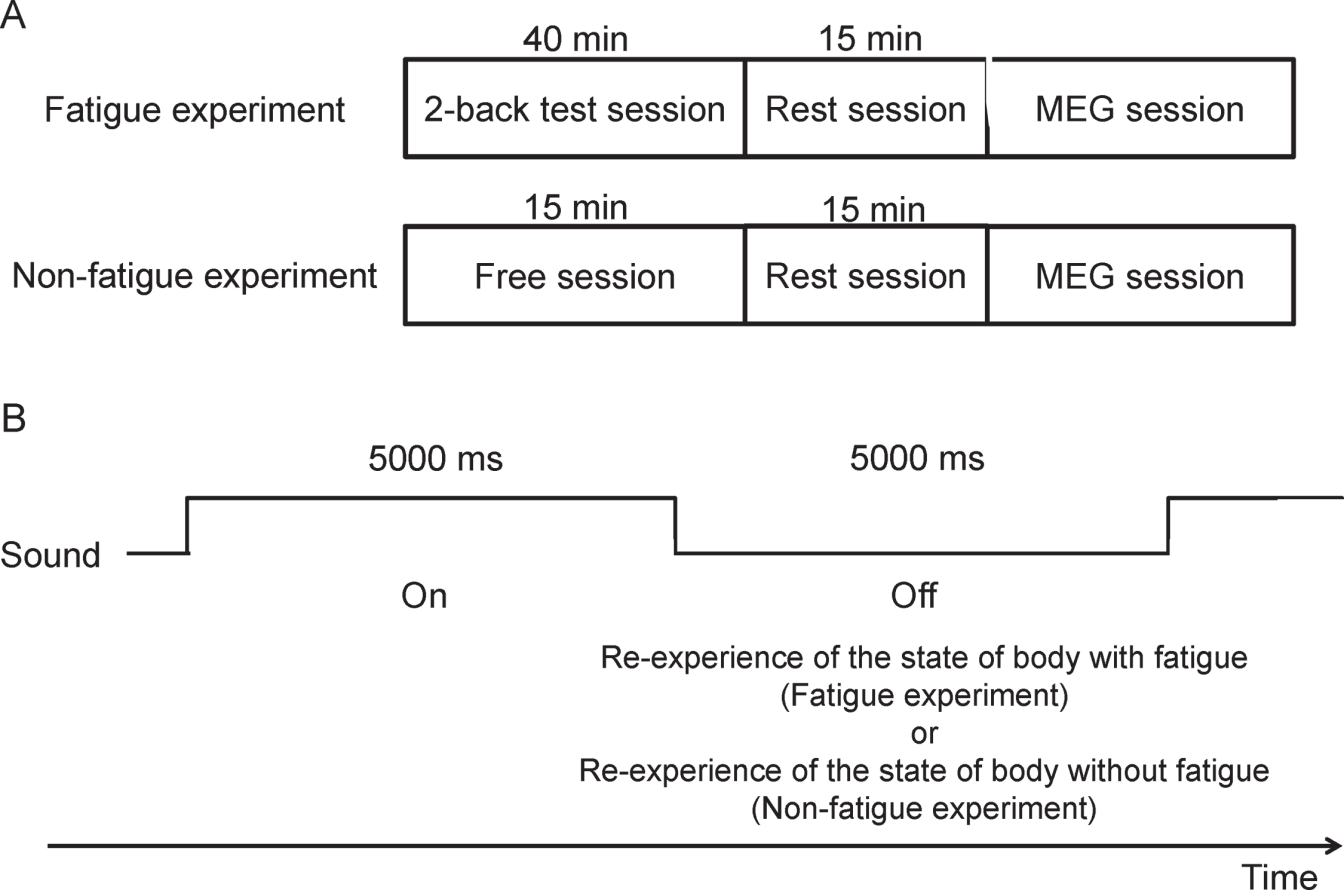
Experimental design. (A) The study consisted of two experiments: a fatigue experiment and a non-fatigue experiment. The two experiments were performed in a crossover fashion and on different days. The fatigue experiment consisted of a 2-back test session for 40 min to induce fatigue sensation, a rest session for 15 min to recover from fatigue, and a magnetoencephalography (MEG) session in which the participants were asked to re-experience the state of their body with fatigue experienced in the 2-back test session. Participants were instructed not to perform the 2-back test in their mind during the MEG session. The non-fatigue experiment consisted of a free session for 15 min in which the participants were instructed to sit quietly so that they did not experience fatigue or any other specific sensation, a rest session for 15 min, and an MEG session in which the participants were asked to re-experience the state of their body without fatigue experienced in the free session. (B) In the MEG sessions, the participants lay on a bed in a magnetically shielded room in the supine position and listened to an auditory cue. The auditory cue was the repetition of a beep with 5-s duration (on phase) followed by a blank with 5-s duration (off phase). They were asked to re-experience the state of their body with and without fatigue, as experienced in the 2-back test and free sessions, respectively, during the off phase of the auditory cue.

The study consisted of two experiments: a fatigue experiment and a non-fatigue experiment (Fig 1A). The two experiments were performed in a crossover fashion and on the same day. We used n-back test as the mental fatigue-inducing task. Since it has been reported that performing 2-back task trials for 30 min induces mental fatigue and mental fatigue sensation [12, 13], the fatigue experiment consisted of a 2-back test session for 40 min to induce mental fatigue sensation, a rest session for 15 min to recover from fatigue, and an MEG session in which the participants were asked to re-experience the state of their body with mental fatigue, as experienced in the 2-back test session. In the 2-back test session, letters were continually presented on the display of a personal computer and the participants had to judge as quickly and as correctly as possible whether the target letter presented was the same as the one that had appeared 2 presentations before. They responded either by pressing the right or the left button when the target was identical to or different from the cue, respectively. The time interval between each trial was 3 s. Participants were instructed not to perform the 2-back test in their mind during the MEG session but to re-experience the state of their body with mental fatigue which had been experienced during the 2-back test session.

The non-fatigue experiment consisted of a free session for 15 min in which the participants were instructed to sit quietly so that they did not experience mental fatigue or any other specific sensation, a rest session for 15 min, and an MEG session in which they were asked to re-experience the state of their body without mental fatigue, as experienced in the free session. In order to re-experience the state of their body not in the rest session but in the free session, the free and 2-back test sessions were performed in the room next to the shield room and the rest session was performed inside the shield room.

In the MEG sessions, the participants lay in a supine position on a bed in a magnetically shielded room and listened to an auditory cue. The auditory cue was the repetition of a beep with 5-s duration (on phase) followed by a blank with 5-s duration (off phase; Fig 1B). They were asked to re-experience the state of their body with and without mental fatigue, as experienced in the 2-back test session and free session, respectively, during the off phase of the auditory cue. The auditory cue was created using OpenSesame software [14]. The level of fatigue sensation that the participants thought to be caused by performing the 2-back task and the level of the fatigue sensation at the beginning of the experiment and before and after the rest session was assessed using a 100-mm visual analogue scale (VAS) ranging from 0 (minimum fatigue) to 100 (maximum fatigue). At the beginning of the first experiment for each participant, the daily level of fatigue was assessed using Japanese version of the Chalder’s fatigue scale [15, 16]. The Japanese version of the Chalder’s fatigue scale consists of 11 questions and the response to each question can be 0 (less than usual), 1 (no more than usual), 3 (more than usual), or 4 (more than usual) during the past several weeks [16].

### MEG recordings

MEG recordings were performed in the MEG sessions using a 160-channel whole-head type MEG system (MEG vision; Yokogawa Electric Corporation, Tokyo, Japan) with a magnetic field resolution of 4 fT/Hz^1/2^ in the white-noise region. The sensor and reference coils were gradiometers with 15.5-mm diameter and 50-mm baseline, and the two coils were separated by 23 mm. The sampling rate was 1,000 Hz and data were high-pass filtered at 0.3 Hz.

### MEG analyses

Before processing the MEG data, the magnetic noise that originated from outside the magnetically shielded room was eliminated by subtracting the data obtained from reference coils using specialized software (MEG 160; Yokogawa Electric Corporation). Epochs of the raw MEG data that included artifacts were visually identified and were excluded from the analyses before averaging. Spatial filtering analysis of the MEG data was performed to identify the changes in oscillatory brain activity that reflected time-locked cortical activities [17–19] caused by re-experiencing the state of the body with and without fatigue. The MEG data were bandpass filtered at 1–4 Hz, 4–8 Hz, 8–13 Hz, 13–25 Hz, and 25–58 Hz by a finite impulse response filtering method using Brain Rhythmic Analysis for MEG software (BRAM; Yokogawa Electric Corporation) to obtain delta, theta, alpha, beta, and gamma signals, respectively. After the bandpass filtering, the location and intensity of the cortical activities were estimated using BRAM, which uses a narrow-band adaptive spatial filtering algorithm [20, 21]. Voxel size was set at 5.0 × 5.0 × 5.0 mm. For each frequency band, the oscillatory power of the MEG data during re-experiencing in the fatigue experiment was calculated relative to the oscillatory power of the MEG data during re-experiencing in the non-fatigue experiment (oscillatory power ratio). To minimize the effects caused by the interruption of the auditory cue, oscillatory power ratio was calculated using MEG data from 2,000 to 4,500 ms after the onset of the off phase of the auditory cue.

These data were then analyzed using statistical parametric mapping (SPM8, Wellcome Department of Cognitive Neurology, London, UK), implemented in Matlab (Mathworks, Natick, MA, USA). The MEG parameters were transformed into the Montreal Neurological Institute T1-weighed image template [22] and applied to the MEG data. The anatomically normalized MEG data were filtered with a Gaussian kernel of 20 mm (full-width at half-maximum) in the x-, y-, and z-axes. To enable inferences to be made at a population level, individual data were summarized and incorporated into a random-effect model [23]. The weighted sum of the parameters estimated in the individual analysis was used to create “contrast” images, which were used for group analyses [23]. The resulting set of voxel values for each comparison constituted a statistical parametric map (SPM) of the t statistic (SPM{*t*}). The SPM{*t*} was transformed to the units of normal distribution (SPM{Z}). Significant differences in the signal between re-experiencing the state without fatigue and that with fatigue were assessed using *t* statistics on a voxel-by-voxel basis [23]. The threshold for the SPM{*t*} of group analyses was set at *P* < 0.05 (familywise-error corrected for multiple comparisons).

### Magnetic resonance (MR) image overlay

Anatomical MR imaging was performed using a Philips Achieva 3.0 TX (Royal Philips Electronics, Eindhoven, The Netherlands) to permit registration of magnetic source locations with their respective anatomical locations. Before MR scanning, five adhesive markers (Medtronic Surgical Navigation Technologies Inc., Broomfield, CO, USA) were attached to the skin of the head: two markers 10 mm in front of the left tragus and right tragus, one marker 35 mm above the nasion, and two markers 40 mm to either side of the marker above the nasion. The MEG data were superimposed on MR images using information obtained from these markers and MEG localization coils.

### Statistical analyses

Values are presented as mean and SD unless otherwise stated. Two-way analysis of variance (ANOVA) with repeated measures was performed to assess the effect of experiment (fatigue and non-fatigue) and time point (beginning of the experiment, before the rest session, and after the rest session) on the subjective level of fatigue. A paired *t*-test with Bonferroni’s correction was used to compare the subjective level of fatigue at the beginning of the experiment to that before and after the rest session. The relation between the change in power between the fatigue and the non-fatigue experiment in Brodmann’s area (BA) 40 and the level of fatigue sensation the participants thought to be caused by performing the 2-back task and the relation between the change in power between the fatigue and the non-fatigue experiment in BA 31 and the daily level of fatigue were evaluated using Pearson’s correlation analyses. All *P* values were two-tailed and values less than 0.05 were considered statistically significant. Statistical analyses mentioned above were performed using the IBM SPSS 21.0 software package (IBM, Armonk, NY, USA). The beta error of the paired t-test were calculated using G*Power 3.1.3 software [24, 25].

## Results

### VAS score

**Fig 2:**
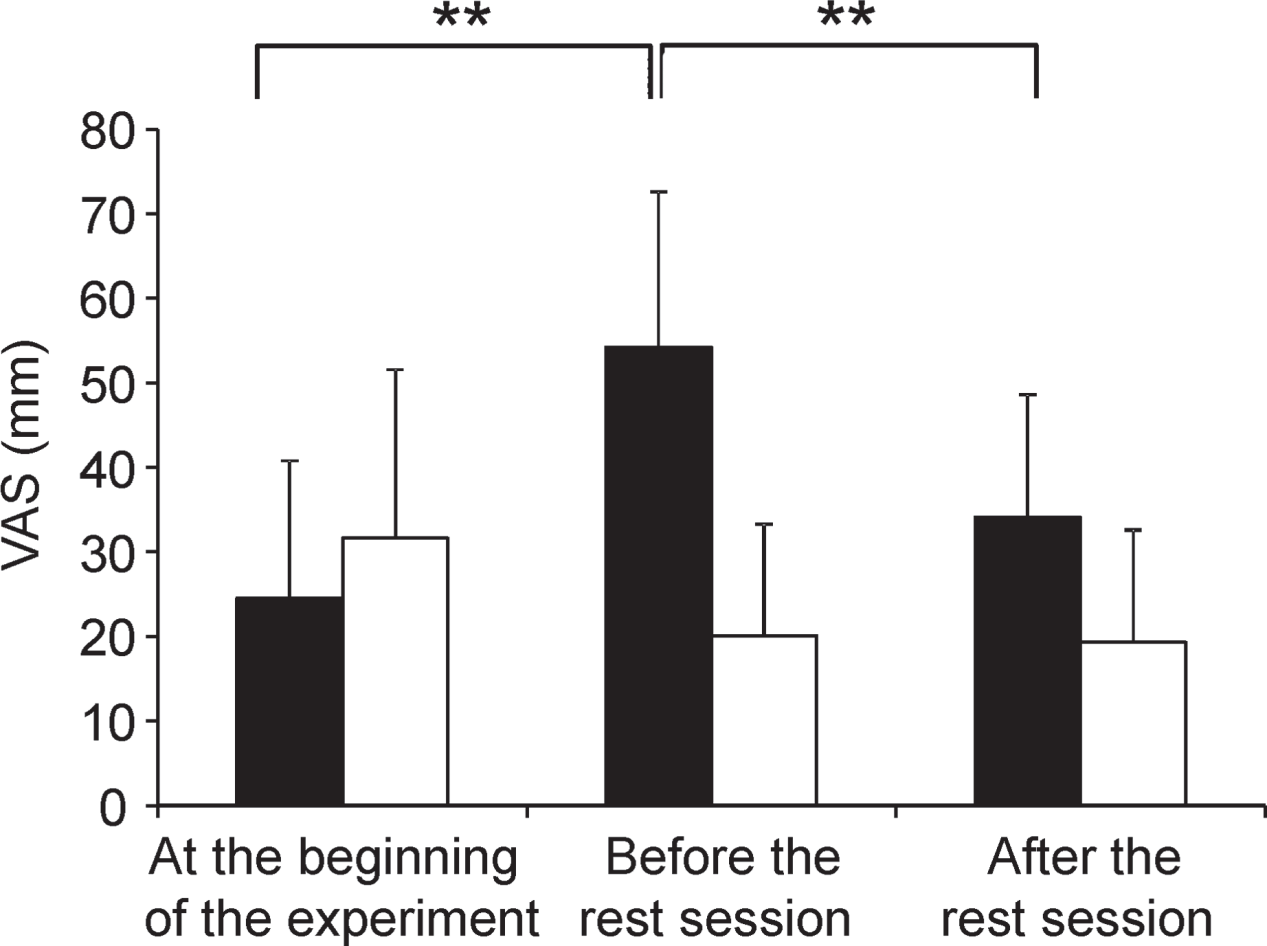
Subjective level of fatigue at the beginning of the experiment and before and after the rest session. Participants were asked to rate the subjective level of fatigue on a 100-mm visual analogue scale (VAS) from 0 (minimum fatigue) to 100 (maximum fatigue). Data are presented as mean and SD. **P < 0.001, paired *t*-test with Bonferroni’s correction.

The subjective level of fatigue assessed by VAS at the beginning of the experiment and before and after the rest session is shown in Fig 2. A two-way ANOVA with repeated measures was performed to compare the subjective level of fatigue across experiments and time points. There was a main effect of time point [F(2, 24) = 9.294, *P* = 0.002], a main effect of experiment [F(1, 12) = 19.688, *P* = 0.001], and a time point × experiment interaction [F(2, 24) = 24.462, *P* < 0.001]. In the fatigue experiment, the VAS score before the rest session was higher than that at the beginning of the experiment (*P* < 0.001, beta error value = 0.00030, paired *t*-test with Bonferroni correction). The VAS score after the rest session was lower than that before the rest session in the fatigue experiment (*P* < 0.001, beta error value = 0.018, paired *t*-test with Bonferroni correction). The mean and SD of the level of fatigue sensation that the participants thought to be caused by performing the 2-back test assessed by VAS were 73.2 and 20.0, respectively.

### Daily level of fatigue

The mean and SD of the daily level of fatigue assessed using the Japanese version of the Chalder’s fatigue scale were 10.2 and 5.4, respectively.

### Spatial filtering analyses of MEG data

**Fig 3:**
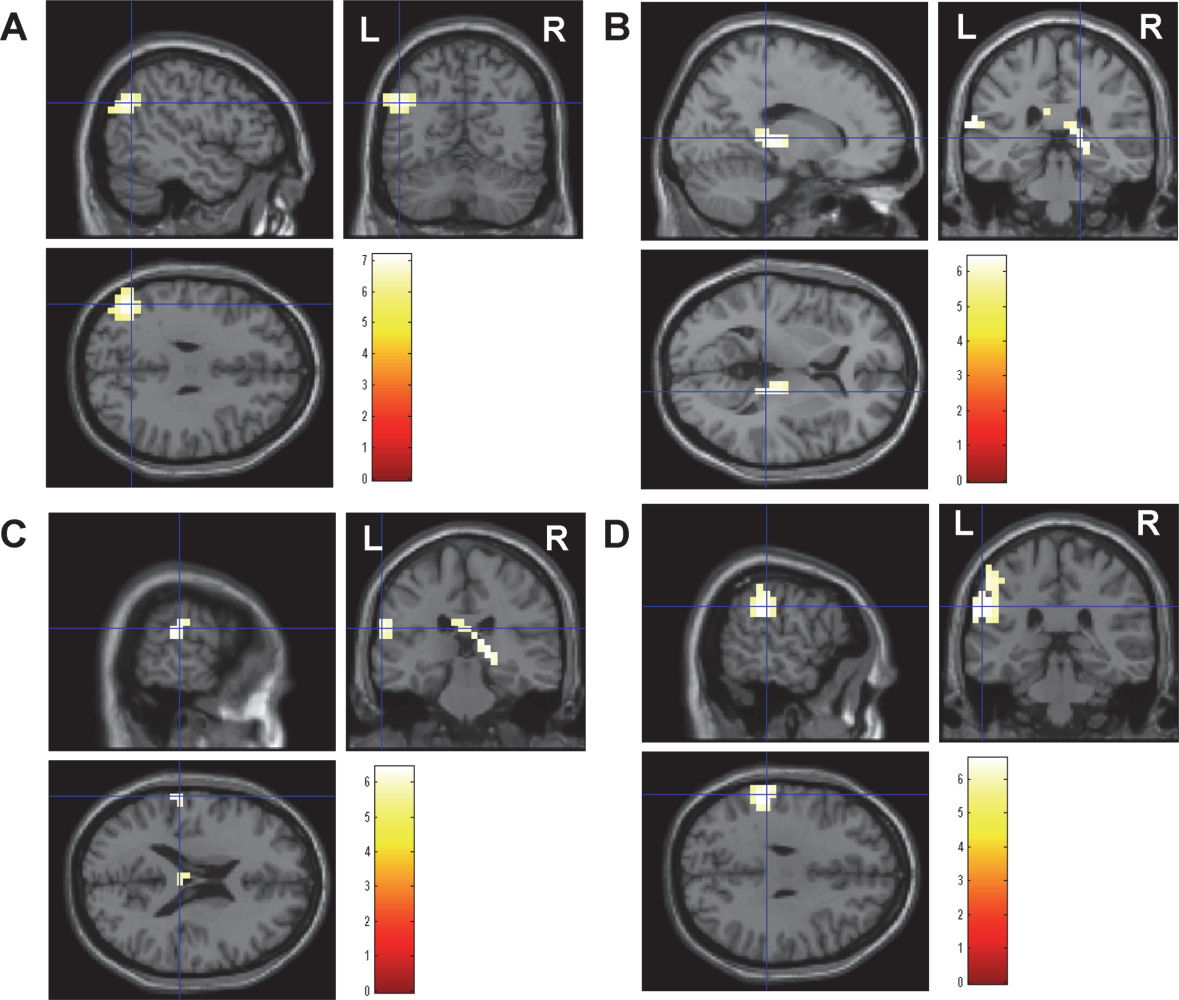
Statistical parametric maps of brain regions where power was lower in the fatigue experiment than in the non-fatigue experiment: the delta band (1–4 Hz) power in the left BA 39 (A), alpha band (8–13 Hz) power in the right pulvinar nucleus (B) and the left Brodmann’s area (BA) 40 (C), and beta band (13–25 Hz) power in the left BA 40 (D). Statistical parametric maps are superimposed on surface-rendered high-resolution magnetic resonance images. The right (R) and left (L) sides are indicated. The color bars indicate t-values. Random-effect analyses of 13 participants, *P* < 0.05, familywise-error corrected for the entire search volumes.

To identify the brain regions activated by re-experiencing the fatigue sensation caused by the 2-back test trials, the oscillatory power in delta, theta, alpha, beta, and gamma frequency bands was compared between the fatigue and non-fatigue experiments. The delta band power in the left BA 39 (Fig 3A), alpha band power in the right pulvinar nucleus (Fig 3B) and the left BA 40 (Fig 3C), and beta band power in the left BA 40 (Fig 3D) were lower in the fatigue experiment than in the non-fatigue experiment (Table 1). The decrease in the beta band power in the left BA 40 was positively associated with the level of fatigue sensation the participants thought to be caused by performing the 2-back task trials (r = 0.669, *P* = 0.012). The decrease in the alpha band power in the right pulvinar nucleus was positively associated with the daily level of fatigue assessed using Chalder’s fatigue scale (r = 0.734, *P* = 0.004).

**Table 1:** Brain regions that showed a greater decrease in oscillatory band power in the fatigue experiment than in the non-fatigue experiment.

Location	Frequency	Side	BA	MNI coordinates (mm)			Z value
				x	y	z	
Superior temporal gyrus	1–4 Hz	L	39	-48	-62	30	4.39
Thalamus (pulvinar)	8–13 Hz	R		17	-32	5	4.15
Postcentral gyrus	8–13 Hz	L	40	-63	-27	20	4.16
Inferior parietal lobule	13–25 Hz	L	40	-58	-32	30	4.21

BA, Brodmann’s area; MNI, Montreal Neurological Institute; L, left; R, right.

x, y, z: Stereotaxic coordinate.

Data were obtained from random-effect analyses. Only significant changes are shown (P < 0.05, familywise error rate).

## Discussion

In the present study, we showed that the delta band power in the left BA 39, alpha band power in the right pulvinar nucleus and left BA 40, and beta band power in the left BA 40 were lower when the participants re-experienced the mental fatigue sensation they had experienced during the 2-back test session than when they re-experienced the state of their body without mental fatigue, as they had experienced during the free session. The decrease in the beta band power in the left BA 40 was positively associated with the level of fatigue sensation the participants thought to be caused by performing the 2-back test. The decrease in the alpha band power in the right pulvinar nucleus was positively associated with the daily level of fatigue assessed using the subjective rating scale, i.e., the Chalder’s fatigue scale [15, 16].

Our findings are in line with previous reports related to the neural mechanisms of autographical recollection and self-induced emotions. In an fMRI study that used a semantic memory decision task as a control condition, activation of the left BA 39 and the left BA 40 was related to re-experiencing past events [26]. In another fMRI study, activation of the left BA 39, left BA 40, and right thalamus was higher during the recollection of emotional autobiographical episodes than during a semantic memory control task [27]. In a H_2_^15^O positron emission tomography study, activation of the right thalamus was observed when anxiety and anger were self-induced [9]. Taking these findings into consideration, the left BA 39, left BA 40, and right thalamus may be related to the re-experiencing and/or recollection of fatigue sensation and the right thalamus may be related to the self-inducing of fatigue sensation required in the process of re-experiencing the fatigue sensation. In the present study, band power was different between the fatigue experiment and the non-fatigue experiment, even though the process of re-experiencing was involved in both experiments. The difference of the band power therefore seems to be related to the contents that were re-experienced, i.e., the fatigue sensation, rather than to the re-experiencing process itself. To support this assumption, the decrease in the beta band power in the left BA 40 was positively associated with the level of fatigue sensation caused by performing the 2-back test.

In the present study, we observed that the alpha band power in the pulvinar nucleus of the thalamus was lower in the fatigue experiment than in the non-fatigue experiment. The thalamus is located deep in the brain and the signal-to-noise ratio of the magnetic signal originating from the thalamus may not be sufficient for reliable source estimation. However, there is accumulating evidence that the source of magnetic signals originating from subcortical regions such as the thalamus, hippocampus, and amygdala can be detected using MEG [28–31]. In fact, electromagnetic activity in the thalamus has been detected in MEG studies using adaptive beamformers to estimate source localization [32, 33]. Together with our finding that the decrease in the alpha band power in the pulvinar nucleus was positively related to the daily level of fatigue, the observed change in the alpha band power in the pulvinar nucleus indicates a role of the pulvinar nucleus in re-experiencing fatigue sensation.

Changes in the oscillatory band powers in the brain regions such as the PCC and the dorsolateral prefrontal cortex were not observed in our present study. These two brain regions have been reported to be activated in a fatigue-provocation condition in the CFS patients compared with that in the healthy participants [6]. In addition, it has just been reported that the PCC is involved in the self-evaluation of the level of fatigue [3, 4]. Taking these into consideration, it seems that the contribution of these brain regions to the neural mechanisms related to re-experiencing fatigue sensation is limited, especially in heathy participants.

There are limitations to our study. First, the number of the participants included in our study was small. To generalize our results, studies with a larger number of participants are needed. Second, neural activity from a deep brain region, i.e., the pulvinar nucleus of the thalamus, was detected. Further studies with other neuroimaging techniques such as fMRI would be beneficial in confirming the neural activity in this brain region. Third, in some brain regions, the observed decrease in band power may have been related to performing the 2-back test. In fact, the BA 40 is involved in the performance of n-back test trials [34]. Thus, we cannot exclude the possibility that the change in band power observed in the present study, particularly the decrease in the alpha and beta band power in the BA 40, was caused because the brain regions involved in performing the 2-back task were activated spontaneously when the participants re-experienced the fatigue sensation experienced in the 2-back task session, even though they were instructed not to perform 2-back test in their mind during the MEG session. To clarify this point, studies with fatigue-inducing tasks which do not require the intensive activation of BA 40, such as physical fatigue-inducing tasks or other types of mental fatigue-inducing tasks are needed. Fourth, since the neural activities caused by re-experiencing the fatigue sensation were assessed only in a single session in each experiment, we cannot estimate the recency effect of memory from our present study. In addition, while the time interval between 2-back test session (or free session) and MEG session was relatively short in our present study, it is of great interest whether the neural activities caused by re-experiencing the fatigue sensation after longer time interval from the 2-back test session (e.g., after one week or later) would be different from those observed in our present study.

In conclusion, we showed that neural activity in the left BA 39, left BA 40, and right pulvinar nucleus were likely related to re-experiencing a mental fatigue sensation. Our findings may help clarify the neural mechanisms underlying fatigue sensation.
